# 2342: How interruptions affect the triage process in the emergency department

**DOI:** 10.1017/cts.2017.263

**Published:** 2018-05-10

**Authors:** Kimberly D. Johnson, Gordon L. Gillespie, Kimberly Vance

## Abstract

OBJECTIVES/SPECIFIC AIMS: The aim of this study was to determining how interruptions affect the triage process. METHODS/STUDY POPULATION: Prospective, observational study, where 118 triage interviews were observed. RESULTS/ANTICIPATED RESULTS: In total, 57% of triage interviews were interrupted. The most common interruption was by other nurses; however, 7% of the interruptions were by the triage nurse themselves. When an interruption occurred during the triage process, 67% of the time the triage nurse would stop the triage assessment and attend to the interrupter. In the interrupted interviews, 17% of the entire triage time was dedicated to addressing interruptions. Some interruptions (ie, additionally staff entering to conduct ECG) had a positive impact by expediting care during the triage process; where other interruptions delayed patient care. DISCUSSION/SIGNIFICANCE OF IMPACT: Interruptions increased the total triage time and contributed to patient treatment delays, as well as led to errors in nursing assessment. Understanding the classifications of triage interruptions and the impact on patient outcomes will allow researchers to develop interventions to mitigate the impact of these interruptions.

